# A Sustainable Business Model for a Neutral Host Supporting 5G and beyond (5GB) Ultra-Dense Networks: Challenges, Directions, and Architecture

**DOI:** 10.3390/s22145215

**Published:** 2022-07-12

**Authors:** Yazan M. Allawi, Alaelddin F. Y. Mohammed, Joohyung Lee, Seong Gon Choi

**Affiliations:** 1 Department of Electrical Engineering, College of Engineering, Princess Nourah Bint Abdulrahman University, Riyadh 84428, Saudi Arabia; ymallawi@pnu.edu.sa; 2 School of Computing, Gachon University, Seongnam 13120, Korea; 3 College of Electrical and Computer Engineering, Chungbuk National University, Chungdae-ro 1, Seowon-Gu, Cheongju 28644, Korea; choisg@cbnu.ac.kr

**Keywords:** neutral host, 5G, DAS, SmC, UDNs, vRAN, RoE

## Abstract

With the deployment of the fifth generation (5G) mobile network systems and the envisioned heterogeneous ultra-dense networks (UDNs), both small cell (SmC) and distributed antenna system (DAS) technologies are required by mobile network operators (MNOs) and venue owners to support multiple spectrum bands, multiple radio access technologies (RATs), multiple optical central offices (COs), and multiple MNOs. As a result, the neutral host business model representing a third party responsible for managing the network enterprise on behalf of multiple MNOs has emerged as a potential solution, mainly influenced by the desire to provide a high user experience without significantly increasing the total cost of ownership (TCO). However, designing a sustainable business model for a neutral host is a nontrivial task, especially when considered in the context of 5G and beyond (5GB) UDNs. In this paper, under an integrated optical wireless network infrastructure, we review how SmC and DAS technologies are evolving towards the adoption of the neutral host business model and identify key challenges and requirements for 5GB support. Thus, we explore recent candidate advancements in heterogeneous network integration technologies for the realization of an efficient 5GB neutral host business model design capable of accommodating both SmC and DAS. Furthermore, we propose a novel design architecture that relies on virtual radio access network (vRAN) to enable real-time dynamic resource allocation and radio over Ethernet (RoE) for flexible and reconfigurable fronthaul. The results from our simulations using MATLAB over two real-life deployment scenarios validate the feasibility of utilizing switched RoE considering end-to-end delay requirements of 5GB under different switching schemes, as long as the queuing delay is kept to a minimum. Finally, the results show that incorporating RoE and vRAN technologies into the neutral host design results in substantial TCO reduction by about 81% in an indoor scenario and 73% in an outdoor scenario.

## 1. Introduction

Today, radio access technologies (RATs) have dramatically advanced from their introduction in the early 1980s. Since then, data traffic generated globally over mobile networks has grown exponentially and is expected to reach up to 160 exabytes per month by 2025 [1]. This explosive growth in mobile data traffic is mainly driven by the increasing prevalence of smartphones and handheld devices and the consequent migration of applications such as video-conferencing, ecommerce, and interactive multimedia services to mobile platforms. However, it comes at a time when mobile network operators (MNOs) are facing an imminent threat of capacity crunch while aiming for a balance between the amount of investment required to upgrade the network infrastructure and the cost per bit paid by users.

Three key approaches have been identified for the fifth generation (5G) mobile network systems to address this problem [2]: the first is to increase cell density by deploying a large number of base stations (BS); the second considers the means by which spectral efficiency can be improved such as enhanced inter-cell interference coordination (eICIC), coordinated multipoint (CoMP) transmission/reception, and massive multiple-input multiple-output (MIMO) techniques; and the third is the introduction of millimeter wave (mmWave) as an entirely new air interface exploiting the underutilized and mostly license-free spectrum found in higher frequency bands. While the latter two options are constrained by theoretical limits and propagation qualities, respectively, network densification is viewed as a relatively more secure investment promising immediate capacity boost through indefinite spatial reuse and closer proximity between the BS and user equipment (UE) [3].

In fact, network densification is not a new concept, where trading off coverage area for capacity gain has been the practice in cellular network planning with the cell radius gradually shortening to reach under a 100 m in some dense urban areas. Thus, it becomes essential to move from dense to ultra-dense networks (UDNs) by further shrinking the cell footprint to a few tens of meters [4]. Consequently, achieving the targets for 5G of a thousandfold system capacity per square meter and tenfold improvement on user experience over its predecessor becomes possible. In addition, this will enable new mobile use cases and business models that are more bandwidth-hungry (such as ultra-high-definition (UHD) video streaming, virtual and augmented reality (VR/AR), virtual marketplaces (VMs), device-to-device (D2D), and vehicle-to-everything (V2X) communications) [1,2,3].

**Figure 1:**
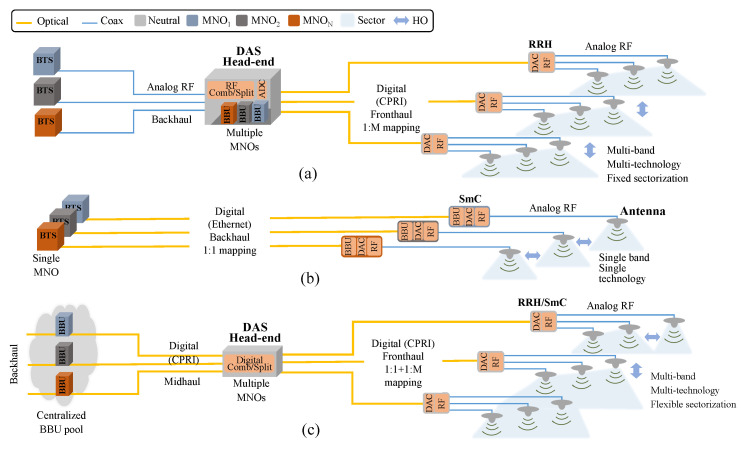
Illustration of (**a**) digital DAS, (**b**) typical SmC, and (**c**) C-RAN-based integration of DAS and SmC in the context of 5GB UDNs.

Small cell (SmC) and distributed antenna system (DAS) are two of the most notable candidates for the realization of UDNs. Both technologies offer the advantages of pinpoint transmission accuracy and improved capacity at a lower power level than their macro cell counterpart, which suffers from poor penetration and low service quality. This is especially the case in indoor environments where more than 80 percent of data traffic is generated [5]. As illustrated in Figure 1a, the baseband processing unit (BBU) in DAS is centralized at a hub location, known as the head-end, which in turn simulcasts the radio frequency (RF) signal to a group of spatially distributed remote radio heads (RRHs) over a high-capacity point-to-multipoint fronthaul (FH) network. Existing DAS equipment is built by design to handle multiple frequency bands, multiple RATs, multiple optical central offices (COs), and multiple MNOs [6]. Indeed, the flexibility and scalability of DAS architecture make it suitable for a wide range of application scenarios. However, the high total cost of ownership (TCO) for DAS has been a major limitation for its prevalence. On the other hand, an SmC (i.e., microcells, picocells, and femtocells), as shown in Figure 1b, is typically comprised of both baseband processing and an RF subsystem in a compact enclosure with a backhaul (BH) link to the base transceiver station (BTS) supporting a single wireless technology for a single MNO [7]. Thus, SmC can be installed as a standalone network or integrated with a macro BS in a heterogeneous network solution more suitable to smaller and scattered coverage areas with much lower upfront capital investment. However, as each SmC has a separate signal source, interference and handover management become more challenging, especially when considered in the context of 5G and beyond (5GB) UDNs.

Recently, a new business model of a third party neutral host responsible for providing access services on behalf of a number of MNOs to a particular location has attracted the attention of many radio access network (RAN) infrastructure providers and venue owners with the desire to provide high user experience without significantly increasing their TCO [5,6]. The need for neutral hosting is further magnified considering the stringent requirements imposed by 5GB UDNs, specifically the high FH capacity, the rising phenomenon of bring your own device (BYOD), and the emergence of new business models such as in-building operators (IBO) and over-the-top (OTT) service providers. Thus, neutral hosting ought to become an indispensable requirement in the design of 5GB RAN solutions.

Despite a growing interest from academia and industry, the research on neutral host design is still in its infancy. In [8], the authors propose an artificial intelligence (AI) and machine-learning (ML) based architectural approach for the design of 5G non-public networks with reduced deployment and operational costs and support for neutral hosting. While different potential use cases were explained, no quantitative analysis or data were provided to demonstrate the feasibility of the proposed architecture in terms of cost efficiency and satisfying 5GB performance requirements. The work in [9] presented a 5G-enabled neutral host framework for citywide deployments utilizing network slicing and RAN virtualization. The framework was deployed in real-world city scenarios and validated considering user experienced data rate, deployment times, and end-to-end (e2e) delay. However, the framework did not offer a cost analysis or address the issue of the 5G delay requirements, even though the experimental results showed an e2e delay exceeding the FH delay budget. To the best of the authors’ knowledge, this paper is the first to investigate the design of a 5GB neutral host for jointly supporting SmC and DAS technologies and to address the issue of resource management along with potential application scenarios. The detailed contributions of the paper are as follows:

1. We identify the key technical challenges facing SmC and DAS, as the most prominent UDN technologies, towards the adoption of the neutral host business model in the context of 5GB requirements and use cases.
2. We explore recent candidate advancements in heterogeneous-network integration technologies to select the ones most suited for the realization of a sustainable 5GB neutral host design capable of accommodating both SmC and DAS based on cost effectiveness and the ability to satisfy the stringent 5GB requirements and diverse use cases.
3. We propose a novel architecture for the sustainable design of a 5GB neutral host business model that relies on a virtual radio access network (vRAN) based on network function virtualization (NFV) to enable real-time dynamic resource allocation and radio over Ethernet (RoE) for flexible and reconfigurable fronthaul.
4. We formulate numerical models for the evaluation of the e2e delay and the amount of TCO reduction performance of the proposed design architecture in contrast to that of its conventional point-to-point fronthauling counterpart and that of RoE-only and NFV-only design variations in real-life indoor and outdoor deployment scenarios.

The rest of this paper is organized as follows: Section 2 provides a comprehensive review of the challenges and requirements entailed in the design of a 5GB neutral host business model. Section 3 presents the proposed design architecture for a sustainable neutral host business model supporting 5GB UDNs. The efficiency of the proposed design is examined in Section 4 considering the e2e delay and TCO reduction over two real-life deployment scenarios, and Section 5 concludes the paper.

## 2. 5GB Neutral Host: Challenges and Requirements

In this section, we discuss in detail how SmC and DAS technologies are evolving towards the adoption of the neutral host business model and identify key technical challenges facing both technologies to support 5GB requirements.

### 2.1. Network Sharing and Multi-Tenancy Support

An essential requirement for the design of neutral host business model is the ability to accommodate multiple MNOs of diverse services and wide range of cellular bands and technologies over a shared RAN infrastructure. Such desire for multi-tenancy capability stems from the fact that rolling out a single physical network has significantly less economical and technical burdens, such as site acquisition, installation cost, spectrum resources, and power consumption, than in the case when each MNO deploys a stand-alone network. When designed carefully, RAN sharing results in a substantial savings on implementation expenditure (IMPEX), capital expenditure (CAPEX), and operational expenditure (OPEX), as well as enabling additional application scenarios and vertical markets as a new source of revenue allowing for a higher return on investment (ROI) [6].

Typically, DAS systems are designed with multi-tenancy support in mind, where many DAS deployments start with a single MNO tenant, and more MNOs are added to the system later on. Thus, it is essential to have a flexible sharing architecture that permits easy additions of MNOs with the ability to handle service and capacity changes. The recent move from legacy analog DAS to a newer breed capable of integrating digital baseband signals to be carried over FH links using the common public radio interface (CPRI) protocol, as illustrated in Figure 1a, has brought additional benefits with respect to efficient sharing such as flexible capacity sectorization and signal routing, dynamic capacity steering, automatic synchronization and delay compensation, and improved scalability. On the other hand, SmC systems have recently adopted the centralized radio access network (C-RAN) architecture and are available with RAN sharing options (i.e., the 3GPP-based multi-operator core network (MOCN) and gateway core network (GWCN) and the non-3GPP but widely used multi-operator RAN (MORAN) [7]). Hence, distinguishing SmC as a capacity-only solution and DAS as a coverage-only solution becomes even harder in the context of 5GB UDNs, where both are expected to expand their application scenarios by coming together in a single integrated solution utilizing the C-RAN architecture as depicted in Figure 1c.

### 2.2. Integration with WiFi

WiFi, as a complement to cellular networks, is a natural choice for traffic offloading considering the abundant bandwidth resources it offers in the unlicensed spectrum and its built-in capability in modern mobile devices. With the portion of traffic offloaded over both carrier WiFi and WiFi-only access points (AP) expected to keep growing to exceed 50 percent of total IP traffic by 2023 [10], the integration of WiFi into the design of a neutral host becomes essential. This is especially true when serving high capacity venues such as airports, university campuses, and enterprises, where the availability of a wide range of WiFi coverage is becoming the norm. However, several challenges need to be addressed in order to make the most of this integration. One key obstacle is traffic steering, which is deciding the time and criteria for switching between WiFi and cellular technologies. With the heterogeneity of RATs as well as the new radio interface operating in high frequencies envisioned for 5GB, such a decision becomes more complicated than the currently practiced approach, which simply relies on detecting a WiFi AP with sufficient signal strength. Hence, other factors including the available RATs, their free resources, loads, and cell visibility, both AP and UE capabilities, radio channel condition, and the number of competing UEs within the same area and their demands should be jointly considered before deciding whether to handover (HO) or not. Given that such a decision is required in real time, and that each RAT would have its own dedicated controller (i.e., WiFi controller (WIC), BS controller (BSC) of 2G, radio network controller (RNC) of 3G, and Mobile Management Entity (MME) of 4G), the responsibilities of traffic steering and HO decision making need to be unified and reassigned to an independent controller that acts on behalf of all these technologies [11]. Another major challenge is coordinating spectrum sharing and interference management, where the difficulty in doing so originates from the fundamental difference in the operational principle of the two technologies. For instance, cellular systems are essentially designed to access the licensed spectrum; thus, they do not need to rely on contention-based MAC protocols to avoid packet collisions, as is the case for WiFi. Hence, mechanisms for the efficient and fair coexistence of cellular and WiFi networks in the unlicensed spectrum need to be regulated [12].

### 2.3. Public Safety Support

Guaranteeing the availability of a broadcast medium across public safety frequencies for supporting first responders is another requirement inseparable from the design of a neutral host. Low frequency bands such as 150 MHz and 450 MHz are currently being used [13]. However, public safety networks seek to enhance their operations for enabling the transmission of high resolution imagery and real-time video footage and utilizing D2D communications by moving to higher spectrum bands such as 700 MHz or 800 MHz [14]. Hence, attention should be paid to the differences in channel characteristics and signal penetration as well as coverage, which includes areas not commonly served by commercial cellular systems such as stairwells and basements. Providing public safety in indoor environments must comply with a number of standards including the national fire protection association (NFPA) code, the international fire code (IFC), and the national electrical manufacturers association (NEMA) [2].

### 2.4. Stringent Fronthaul Requirements

While resorting to heterogeneous UDNs can significantly improve the capacity of cellular networks to the level desired for 5GB, the consequent increase in the amount of offloaded traffic as well as the adoption of C-RAN architecture in the neutral host design would result in stringent requirements on the FH network segment in terms of capacity, latency, and cost efficiency. Until recently, FH deployments mainly considered CPRI protocol, which is particularly designed to transport sampled radio waveforms. However, CPRI has a number of drawbacks [15]:

- Frames are transmitted at a constant bit rate regardless of user activity and actual data traffic, where the CPRI signal is typically 50 times the user bit rate.
- CPRI allows only one-to-one (1:1) mapping between RRH and BBU, whereas it is desirable to have many-to-many (M:M) mapping for flexible RRH–BBU affiliation.
- The CPRI line rate increases proportionally to the carrier bandwidth and the number of antennas and sectors considered in the cell configuration, such that in order to achieve the 20 Gbps system capacity required by 5G for an 8 × 8 MIMO 3-sectors cell site and using 256 QAM, the resulting CPRI line rate per RRH grows to a staggering 560 Gbps.
- There is a strict e2e delay to meet HARQ requirements allowing for 100–250 μs only on the FH link, while jitter needs to be maintained in nanoseconds scale.

Hence, enhancements to existing FH interfaces as well as possible alternatives are currently being investigated in order to cope with 5GB requirements and reduce deployment costs. This has led CPRI cooperation to issue an enhanced version of CPRI (eCPRI) [16] that introduces a new functional split within the physical layer targeting a tenfold reduction in required bandwidth and the use of packet-based transport technologies such as ethernet to carry CPRI traffic. This is achieved by performing the cyclic prefix, the Fast Fourier Transform with guard band subcarriers, and demapping of the resource blocks at the RRH, which results in a large reduction in the amount of overhead data. In addition, the impact and potential gains of other functional splits beyond the scope of the physical layer and the utilization of RoE as a replacement for CPRI are currently being investigated by standardization bodies including the IEEE 1914 next-generation FH interface (NGFI) working group with two active projects, P1914.1 for packet-based FH transport networks, and P1914.3 for RoE encapsulations and mappings and the IEEE 802.1CM time-sensitive networking (TSN) task group with 802.1Qbu for frame preemption, 802.1Qbv for traffic scheduling, and 802.1Qca for path control and reservation [17].

### 2.5. Automatic Sectorization and Coverage Control

Alongside cell site densification, sectorization is another straightforward technique to increase network capacity. Automatic sectorization allows any number of sectors to be flexibly configured depending on the venue requirements and time of day, which can be a simple one large cell requiring no HO between antennas or divided into more sectors, each representing a different simulcast domain for further capacity gains. However, beyond a certain number of sectors, the additionally introduced intersector interference and the increased number of HO zones become hard to control [18]. Hence, deciding the sectorization number and coverage needs to be optimized in real time such that the resulting total venue capacity is maximized. Automatic sectorization is made possible by relying on a reconfigurable antenna design, where the azimuth beamwidth and tilt can be controlled by a tunable reflector.

### 2.6. Dynamic Resource Allocation

A key feature required in a 5GB neutral host is the capability to react to actual traffic demands by the dynamic allocation of system capacity whenever it is needed. This becomes apparent when considering the tidal effect experienced by multi-tenant highrise buildings and citywide multi-venue deployment scenarios, where traffic loads of different cells vary significantly over time. For example, the traffic demands of enterprise offices and commercial buildings peak during day time and recede at night time, whereas it is the opposite for residential traffic. Therefore, efficient resource utilization techniques are required for enabling statistical multiplexing gains at inter-service, inter-MNO, and inter-venue levels, thus achieving the desired TCO reduction and power saving. For this, network slicing solutions based on software-defined networking (SDN) and network function virtualization (NFV) are considered to enable flexible creation of performance- and cost-customized logical networks over a shared physical infrastructure [5].

In addition, the design of a 5GB neutral host can benefit from utilizing ML algorithms and techniques to facilitate network automation and handle the monitoring, control, and management network functions more efficiently, which consequently improves the dynamic resource allocation, especially for complex deployment scenarios and novel 5GB network use cases with high environmental uncertainty [19,20]. The work in [8] provides details on how AI/ML functionality is exploited alongside NFV to support neutral host RAN sharing and achieve a holistic network resource optimization.

### 2.7. Zero-Touch Security

With the support of multiple MNOs and the coexistence of SIM and non-SIM based devices over cellular and WiFi networks, seamless authentication becomes a concerning issue for the neutral host design. This is especially true for application venues with high security and privacy requirements such as financial institutions, government offices, and healthcare facilities. For this, security features for accessing a 3GPP evolved packet core (EPC) have been defined in Release 15 [21] by distinguishing two cases, ‘Trusted’ in which the MNO assumes that switching to non-3GPP access is protected by the 3GPP network without the need for a separate authentication process and ‘Untrusted’ when the 3GPP network requires an additional authentication process through the IP tunneling mechanism of IPSec protocol. Similarly, Wi-Fi Alliance has recently issued the specifications for Hotspot 2.0 for automatic secure authentication and device discovery and selection. Hotspot 2.0 allows the decision of switching the access network to be device driven, where compliant SIM and non-SIM devices can connect seamlessly without the need for user intervention [22].

## 3. Design Architecture for Sustainable 5GB Neutral Host Business Model

As discussed in the previous section, the current implementation option for 5G mobile networks based on C-RAN architecture brings a significant TCO reduction by eliminating the need for bulky RF processing modules at the head-end as well as enabling dynamic resource allocation through tighter inter-cell coordination, while at the same time, it imposes stringent requirements on the fronthaul link [23]. In particular, when considered in the context of a neutral host supporting 5GB UDNs, even the TCO reduction merit of a C-RAN based design, such as the one depicted in Figure 1c, becomes elusive given that baseband only accounts for a third of BS’s CAPEX. In addition, a C-RAN-based design would have a limited capability to adapt to changing RF distribution requirements such as tidal traffic patterns. In contrast, a vRAN based architecture, where certain network functions are decoupled from network hardware and implemented on general purpose processor (GPP) servers, runs the centralized functions over a pool of virtual machines that can be allocated on demand by a hypervisor, thus eliminating the need for overprovisioning and enabling achievement of the desired level of TCO reduction [24].

**Figure 2:**
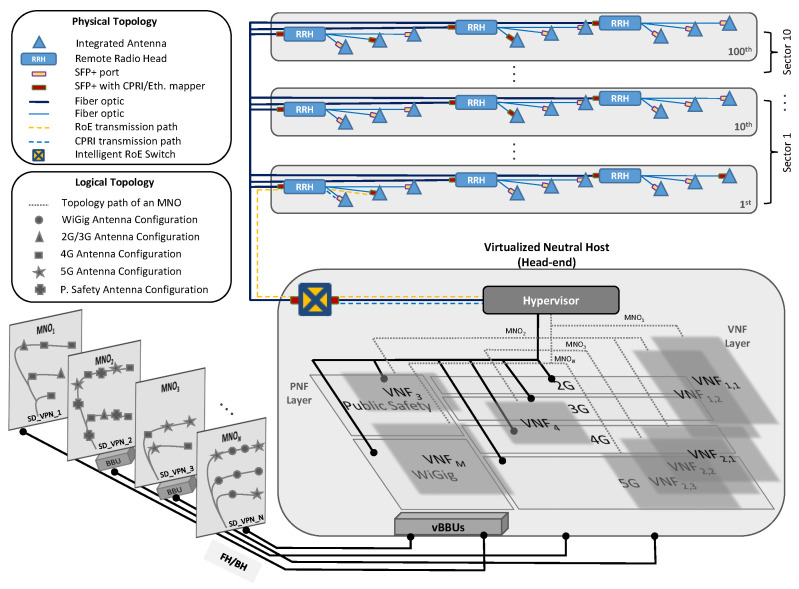
Illustration of the proposed 5GB neutral host design architecture utilizing RoE and vRAN technologies.

Accordingly, in this section, we present a novel design architecture for a 5GB neutral host business model capable of accommodating both SmC and DAS by utilizing vRAN architecture to enable real-time dynamic resource allocation and RoE for a flexible and reconfigurable FH. As illustrated in Figure 2, the proposed design architecture consists mainly of two components: (1) the RRHs, which are given the capabilities of interfacing with multi-technology and multi-band integrated antennas, differentiating the incoming traffic into one-to-one and one-to-many based on the configuration of the intended destination that can be an SmC or DAS and deciding whether the incoming traffic should be encapsulated in RoE frames or de-encapsulated and sent as CPRI frames to account for different functional splits; (2) the virtualized neutral host at the head-end, which is capable of hosting multiple MNOs regardless of their various deployment plans (i.e., which sectors, floors, and antennas are to be served and which technologies and bands are to be dynamically configured in response to spatiotemporal traffic demands). Based on the deployment plan and which functional split is considered by each MNO, the head-end is fed with an FH or BH signal while allowing for the allocation of BBUs, which are responsible for baseband signal processing and base station control, to be off-premise or as a pool of virtualized BBUs (vBBUs) that can be instantiated at the head-end whenever needed.

Within the virtualized neutral host comes the hypervisor, which decouples the physical network resources from the virtual networks to enable multiple tenant MNOs to share the same physical infrastructure, each with its own topology and addressing scheme. In addition, the hypervisor assumes the responsibility of defining the required virtual network functions (VNFs), VNF chaining, and the mapping of each tenant MNO to its chains of VNFs. An example is given in Figure 2 that considers a 100-story building with 10 sectors run by an IBO. Here, $MNO_{1}$ contracts with the IBO to provide 2G, 3G, and 4G access to specific antennas in different sectors of the building. Accordingly, the hypervisor instantiates $VNF_{1}$ and maps it to $MNO_{1}$ through the route $SD\_VPN_{1}$ as a BH signal given that $MNO_{1}$ has decided to utilize the vBBUs at the head-end. $MNO_{2}$ on the other hand, contracts for the same $VNF_{1}$ resources in addition to 5G access over a different set of antennas in different sectors and public safety access to cover the whole building. The hypervisor recognizes that $VNF_{1}$ has been already mapped to $MNO_{1}$, and thus slices $VNF_{1}$ into $VNF_{1,1}$ and $VNF_{1,2}$ to be shared between the two MNOs. In addition, the hypervisor will instantiate a new $VNF_{2}$ for the 5G resource and $VNF_{3}$ for public safety and route the traffic of the $VNF_{1,2}$, $VNF_{2}$, and $VNF_{3}$ associated with $MNO_{2}$ back to its off-premise BBUs through $SD\_VPN_{2}$ as an FH signal. The hypervisor follows the same procedure whenever a new MNO is added or when the deployment plan of an already existing MNO incurs any modifications.

## 4. Performance Evaluation

**Figure 3:**
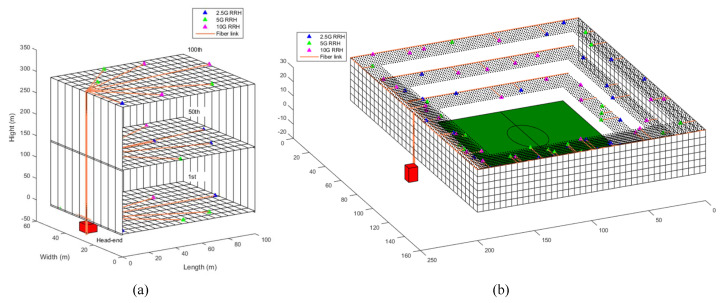
Deployment scenarios of the proposed 5GB neutral host architecture. (**a**) One-hundred-story commercial building. (**b**) Stadium.

**Figure 4:**
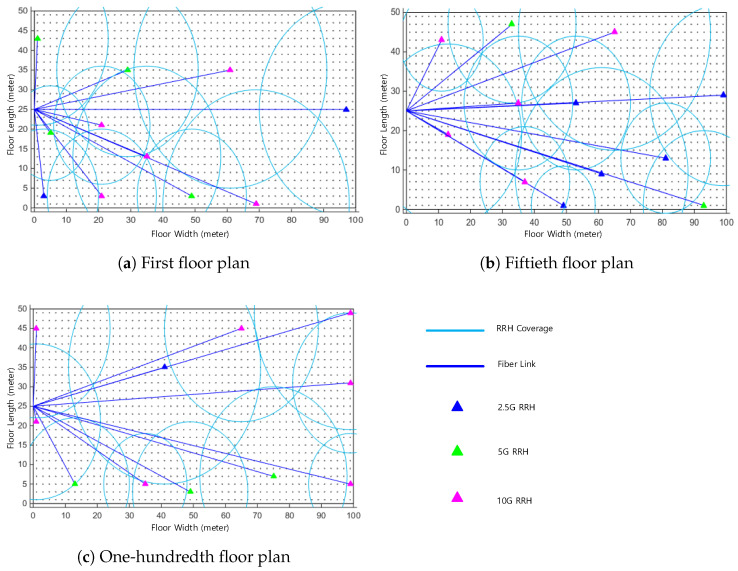
Illustrative example of the RRHs’ random distribution for the 100-story commercial building scenario.

**Figure 5:**
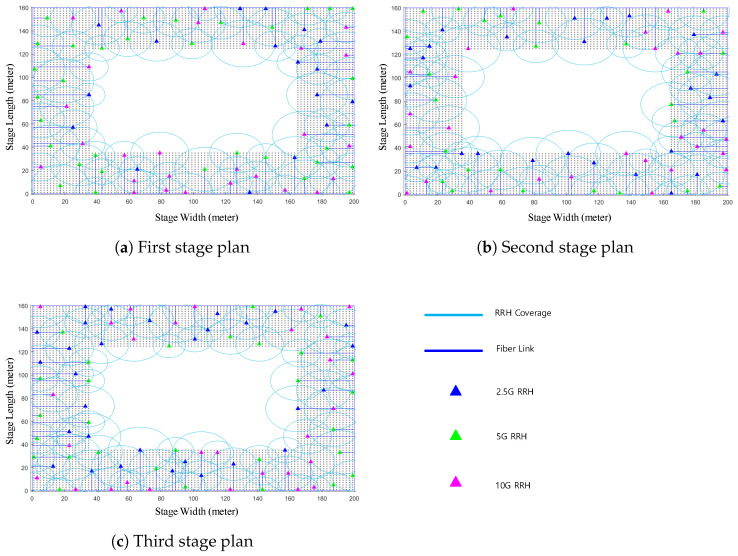
Illustrative example of RRHs’ random distribution for the stadium scenario.

In this section, the efficiency of our proposed 5GB neutral host design architecture is examined in terms of the e2e delay and the amount of TCO reduction. For this, we used MATLAB to implement our proposed architecture for the design of a 5GB neutral host of two real-life deployment scenarios, namely a 100-story commercial building representing an indoor scenario and a stadium representing an outdoor scenario. In the first scenario, illustrated in Figure 3a, each floor of the 100-story building, which has an area of 100 × 60 $m^{2}$, was partitioned with a granularity of 1 $m^{2}$, RRHs with an FH capacity that could be either 2.5, 5, or 10 Gbps, and a coverage radius selected between 10 and 30 m randomly located over the floor plane until full coverage was achieved. Figure 4 gives an example of how the random distribution of RRHs was performed for the 1st, 50th, and 100th floors of the commercial building, which resulted in a total number of 1112 RRHs required to cover the whole building. For the second deployment scenario, depicted in Figure 3b, the stadium had an area of 200 × 160 $m^{2}$ and consisted of three stages. We followed the same procedure as in the first scenario to partition each stage and randomly distributed the RRHs as illustrated in Figure 5. However, in this case, we selected the radius for the coverage area of an RRH between 10 and 20 m to account for the fact that the density of mobile users at a stadium would be higher than that in a commercial building. Accordingly, the resulting total number of RRHs required to cover the stadium was 284.

### 4.1. e2e Delay

Investigating the e2e delay becomes necessary for our proposed 5GB neutral host design architecture in order to verify the applicability of utilizing switched RoE as a replacement for CPRI in the FH segment. Hence, we first formulated the delay incurred by conventional point-to-point (P2P) fronthauling $D_{P2P}$, which corresponded to the 1:1 mapping between each pair of RRH and BBU in the C-RAN architecture to benchmark the RoE delay $D_{RoE}$, where $D_{P2P}$ represents the lowest possible delay consisting of two deterministic components, the propagation delay $D_{P}$ and the transmission/reception delay $D_{TRx}$, as expressed in Equation (1). $D_{RoE}$ is formulated in Equation (2) and accounts for the encapsulation/decapsulation delay $D_{CAP}$, switching delay $D_{SW}$, queuing delay $D_{Q}$, and the number of RoE switches S. Here, all delay components were deterministic except for $D_{Q}$, which varied based on traffic status.

(1) $$D_{P2P}=D_{P}+D_{TRx},$$

(2) $$D_{RoE}=D_{P}+2D_{CAP}+S\left(D_{SW}+D_{TRx}+D_{Q}\right),$$

where, in our simulations, $D_{P}$ = 5d, such that d is the distance between RRH and BBU in km, $D_{TRx}$ was calculated as 7.2 μs for a maximum transmission unit MTU = 9000 byte and as 1.2 μs for MTU = 1500 byte, $D_{CAP}$ = 8 MTU/CPRI rate with the CPRI rate selected from {2.5, 5, 10} Gbps, $D_{SW}$ was calculated as 3 μs when using store-and-forward (SAF) switching and as 10 μs for cut-through (CT) switching, and $D_{Q}$ was selected from {0, 20, 40, 60, 80} μs. In addition, we set the FH delay budget to 250 μs, which if exceeded results in throughput degradation, based on LTE-A/FDD requirements [25].

**Figure 6:**
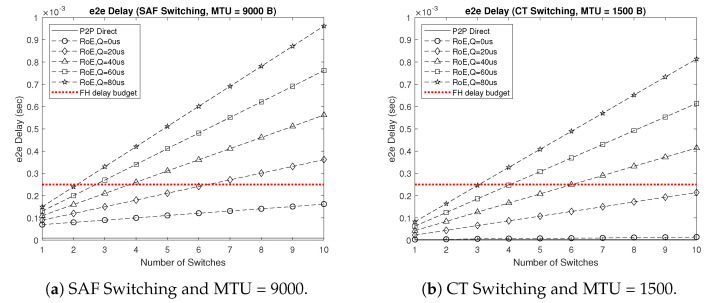
e2e delay results of the proposed 5GB neutral host design architecture for the 100-story-building deployment scenario.

**Figure 7:**
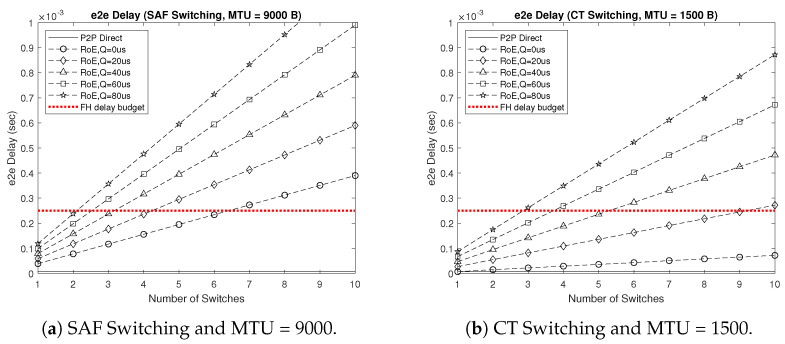
e2e delay results of the proposed 5GB neutral host design architecture for the stadium deployment scenario.

Figure 6 shows the e2e delay results of our proposed 5GB neutral host design architecture for the 100-story building deployment scenario depicted in Figure 3a. Here, we considered the worst case of using SAF switching and MTU = 9000 and the best case of relying on CT switching and setting MTU = 1500 instead. Comparing the results of the two cases, we first noticed the benefit of using CT switching with a smaller size MTU, which resulted in a performance comparable to that of P2P fronthauling, assuming no queuing delay. However, as soon as a queuing delay was introduced, we saw a drastic increase in the e2e delay, which limited the number of switches that could be accommodated in a switched RoE setup without violating the 250 μs delay budget to only three in the best case scenario, when the queuing delay was set as high as 80 μs. Figure 7 shows a higher e2e delay for the stadium deployment scenario resulting in further limiting the number of switches allowed along the FH route. This was evident from the results of both deployment scenarios in the worst case of SAF switching and MTU = 9000 with the assumption of no queuing delay, where accommodating more than ten switches was still possible in the commercial building scenario while maintaining the delay below 250 μs; whereas, it was limited to only six switches for the stadium scenario. This was mainly due to the increased average distance between the RRHs and BBUs in the stadium scenario compared to that of the commercial building. Hence, the utilization of switched RoE in the FH segment of our proposed 5GB neutral host architecture is in fact feasible as long as the queuing delay is kept to a minimum, which can be achieved based on 802.1Qbv traffic scheduling and 802.1Qbu frame preemption. With the merits of enabling a flexible functional split and reconfigurable FH, RoE represents an attractive deployment option as a replacement for the CPRI protocol.

### 4.2. TCO Reduction

To investigate the cost effectiveness of our proposed 5GB neutral host design architecture, the amount of TCO reduction made possible by the utilization of RoE and NFV technologies was examined by conducting a comparison with the TCO resulting from the P2P fronthauling of C-RAN as a benchmark and that of RoE-only and NFV-only over the 100-story commercial building and the stadium deployment scenarios discussed earlier. To accomplish this, we relied on the formulation of TCO as expressed in Equation (3) with the notations used hereinafter summarized in Table 1 [26,27,28].

(3) $$TCO=\sum_{i=1}^{I}\frac{{\left(CAPEX+IMPEX\right)}_{i}}{AP}+\sum_{i=1}^{I}OPEX_{j}.$$

**Figure 8:**
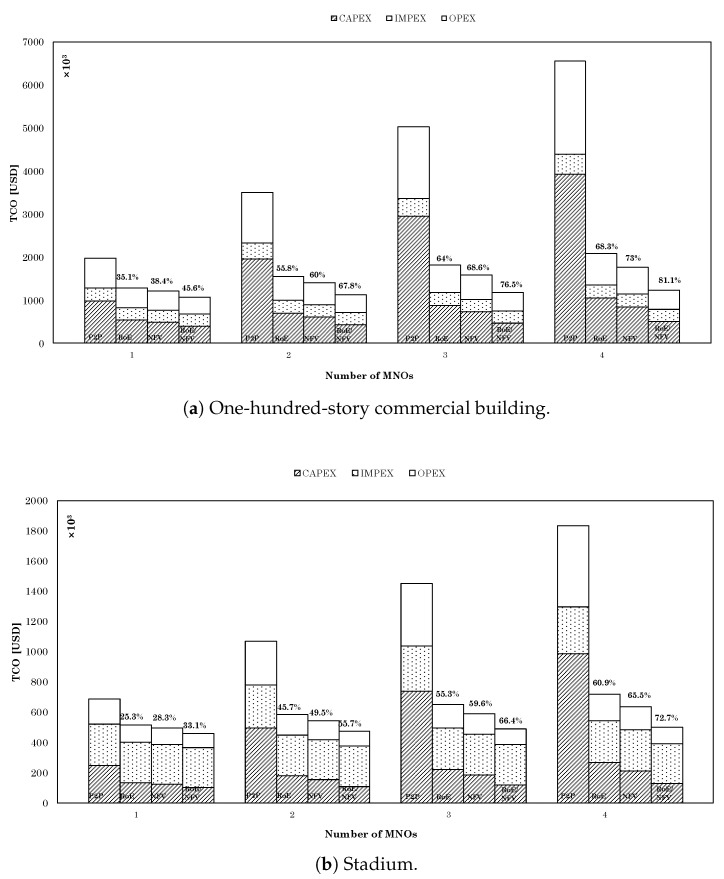
TCO results of the proposed RoE/NFV-based 5GB neutral host design architecture versus P2P, RoE-only, and NFV-only over the deployment scenarios.

Here, CAPEX was calculated differently for the deployment options of P2P, RoE-only, NFV-only, and that of the proposed RoE/NFV, as given in Equations (1)–(7), respectively; whereas, the generic formulations for IMPEX and OPEX are given in Equations (8) and (9), respectively.

(4) $$\begin{array}{cc} CAPEX_{P2P}=MNO[N(C_{BBU}+C_{RRH_{r}}+ & \\ & 2C_{SFP}+C_{fiber}\left(d_{FH}+d_{BH}\right)+f_{n}*C_{Host})], \end{array}$$

(5) $$\begin{array}{c} CAPEX_{RoE}=MNO\left[N\left(\frac{C_{BBU}}{G_{MUX}}+f_{n}*C_{Host}\right)+d_{BH}*C_{fiber}\right]+S*C_{SW}+N\left(C_{RRH_{r}}\right)\\ +\frac{C_{SFP(G_{MUX}+1)}}{G_{MUX}}+d_{FH}*C_{fiber}, \end{array}$$

(6) $$\begin{array}{c} CAPEX_{NFV}=MNO*N\left[C_{BBU}+2C_{SFP}+f_{n}*f_{m}*C_{Host}\right]+d_{BH}*C_{fiber}+N\left(C_{RRH_{r}}\right)\\ +d_{FH}*C_{fiber}, \end{array}$$

(7) $$\begin{array}{c} CAPEX_{RoE/NFV}=MNO*N\left[\frac{C_{B}BU}{G_{M}UX}+f_{n}*f_{m}*C_{Host}\right]+d_{BH}*C_{fiber}+S*C_{SW}\\ +N(C_{RRH_{r}}+\frac{(C_{SFP}\left(G_{MUX}+1\right)}{G_{MUX}}+d_{FH}*C_{fiber}), \end{array}$$

(8) $$IMPEX=f_{imx}*CAPEX+d_{BH}*C_{Trench},$$

(9) $$\begin{array}{c} OPEX=C_{site}+C_{BH}*BW+C_{run}+f_{mnt}*CAPEX+N*C_{pwr_{R}}, \end{array}$$

where *N* represents the number of RRH to BBU connections, MNO is the number of collocated MNOs, BW is the amount of bandwidth the neutral host consumes in the BH segment, and $d_{BH}$ and $d_{FH}$ are the length of BH and FH segments, respectively. Figure 8 shows the TCO analysis results when setting the delay budget to 250 μs, assuming a queuing delay of 40 μs, and the use of CT switching with MTU = 1500 byte.

Examining the results of the 100-story-building deployment scenario depicted in Figure 8a, we notice that even when the neutral host was serving a single MNO, the utilization of RoE-only in the fronthaul segment allowed for more than a 35% reduction in TCO when compared to that of the conventional P2P deployment solution. This is mainly due to the merit of RoE in enabling flexible FH/BH configuration and taking advantage of different functional splits to achieve the desired statistical multiplexing gain. We can also observe that the utilization of NFV-only had more influence on TCO reduction with a 3.3% improvement over that of RoE-only. By combining RoE and NFV in our proposed architecture, the amount of TCO reduction increased to 45.6%. As we increased the number of MNOs served by the neutral host, further TCO reduction was achieved, where having four MNOs in case of the proposed RoE/NFV, the TCO reduction reached 81.1% when compared to the P2P deployment solution in which each of the MNOs would serve their users over a standalone network. The results for the stadium deployment scenario depicted in Figure 8b showed a similar pattern to that of the commercial building, where again a considerable improvement was achieved by the utilization of RoE and NFV in our proposed architecture, such that the TCO was improved by 72.7% in the case of serving four MNOs. Comparing the results of the two deployment scenarios, we notice a lower TCO in total as well as higher values of IMPEX at the expense of CAPEX and OPEX, which is due to the fact that fewer RRHs are required to provide full coverage for the stadium deployment scenario than in the case of the commercial building, as explained in the previous section.

## 5. Conclusions and Future Work

While mobile operators are racing towards 5G rollout with rising anxiety about the right choice of investment, the neutral host business model has emerged as an attractive solution for its evident cost effectiveness. At the same time, SmC and DAS, representing the most prominent solutions for the realization of 5GB UDNs, are coming together in a single integrated solution based on the neutral host model. In this paper, we discussed in detail the challenges and requirements for designing a neutral host capable of supporting 5GB UDNs and use cases. Based on recent advancements in wireless transmission and network integration, we presented a sustainable design architecture of a neutral host business model supporting 5GB UDNs, which utilized vRAN to enable real-time dynamic resource allocation and RoE for flexible and reconfigurable FH.

The simulation results showed that the use of RoE as a replacement for the CPRI protocol in the FH segment was efficient in terms of satisfying the e2e delay requirements set for 5G. Moreover, our TCO analysis conducted for the case of RoE-only and that of NFV-only highlight the advantage that each of them brings separately to the design of neutral host business model in terms of TCO reduction. By enabling flexible functional split and consequently reducing bandwidth requirements in the FH/BH segment, an RoE-only design contributes to the reduction in TCO by more than 68% in an indoor scenario and 60% in an outdoor scenario. In comparison, an NFV-only design further reduces the TCO by about 3% due to the ability of creating customized logical networks over a shared physical infrastructure. While these results are substantial in view of their conventional P2P fronthauling counterpart, the combination of both technologies in a single solution as suggested in our proposed RoE/NFV-based 5GB neutral host design architecture is far more rewarding given a TCO reduction of about 81% in an indoor scenario and 73% in an outdoor scenario.

Since the problem of designing neutral host networks is relatively new, several extensions from our work are possible. For instance, we would consider in our future work the design of multi-tenant highrise building and citywide multi-venue deployment scenarios for which special attention is required to the capacity dimensioning issue to account for the tidal effect. In this context, our proposed 5GB neutral host design architecture can be extended by adopting ML algorithms as discussed in Section 2.6 and investigating its influence on TCO reduction in terms of management costs and total energy consumption.

## Figures and Tables

**Table 1:** Simulation parameters.

Symbol	Description	Value
AP	Amortization period	5 years
$C_{BBU}$	Cost of a single BBU	USD 00
$C_{RRH_{r}}$	Cost of a single RRH of FH capacity 7 2.5/5/10 Gbps	USD 1/1.5/2 k *
$C_{SFP}$	Cost of a single SFP port	USD 78
$C_{fiber}$	Cost of fiber cable per km	USD 160
$C_{Trench}$	Cost of trenching per km	USD 130 k
$C_{Host}$	Cost of neutral host equipment	USD 12.8 k
$C_{SW}$	Cost of a single RoE switch	USD 2930
$C_{BH}$	Cost for backhauling	USD 1170
$C_{site}$	Annual site leasing cost	USD 3300
$C_{run}$	Annual total cost of running the site	USD 1000
$C_{pwr_{R}}$	Power consumption cost associated with coverage radius R of 10/20/30 m	USD 50/100/150
$G_{MUX}$	Multiplexing gain factor	4
$f_{n}$	Coefficient of number of RRH to BBU connections	0.05
$f_{m}$	Coefficient of number of MNOs	0.05
$f_{imx}$	Coefficient of implementation cost	0.25
$f_{mnt}$	Coefficient of site maintenance cost	0.8

* k denotes (×1000) whenever it appears in cost values.
